# Supplementary material for: Changes in intraocular pressure before, during, and after playing Thai Traditional, Thai Folk, and Western wind instruments
Source: PLoS One. 2025 Oct 23;20(10):e0333533. doi: 10.1371/journal.pone.0333533 (PMC12548883; doi:10.1371/journal.pone.0333533)

Supporting information

S1 Fig. The musical scale used by all musicians and the timepoint of IOP measurement in each eye.

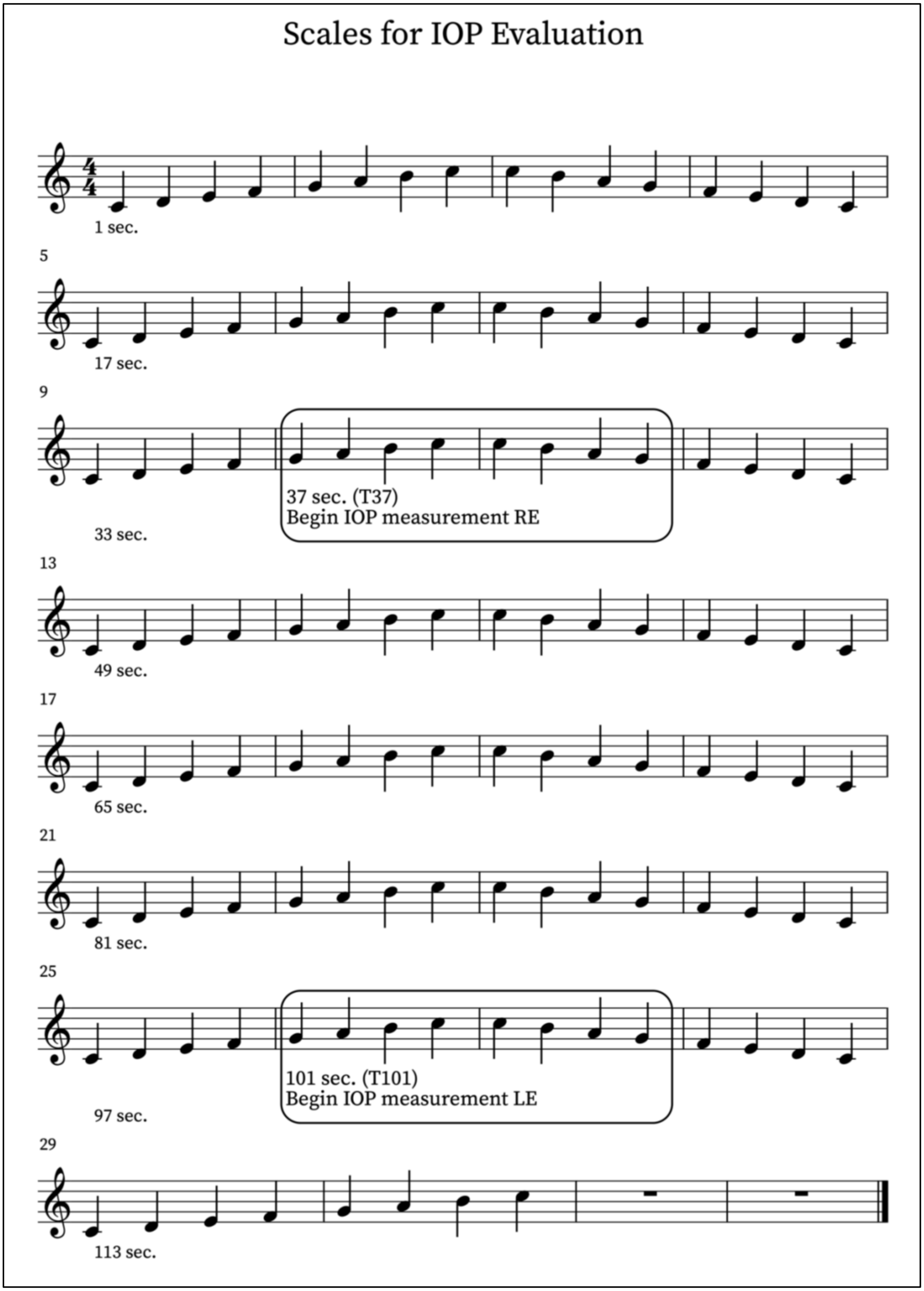

Supplement: S1 Fig — (PDF) [file pone.0333533.s001.pdf]
