# Supplementary material for: Changes in intraocular pressure before, during, and after playing Thai Traditional, Thai Folk, and Western wind instruments
Source: PLoS One. 2025 Oct 23;20(10):e0333533. doi: 10.1371/journal.pone.0333533 (PMC12548883; doi:10.1371/journal.pone.0333533)
Supplement: S2 Fig — (PDF) [file pone.0333533.s002.pdf]

Supporting information

S2 Fig. Instruments used in each group and participants' postures during IOP measurement across all groups.

Group 1: Western (WT) wind instruments; saxophone and trumpet

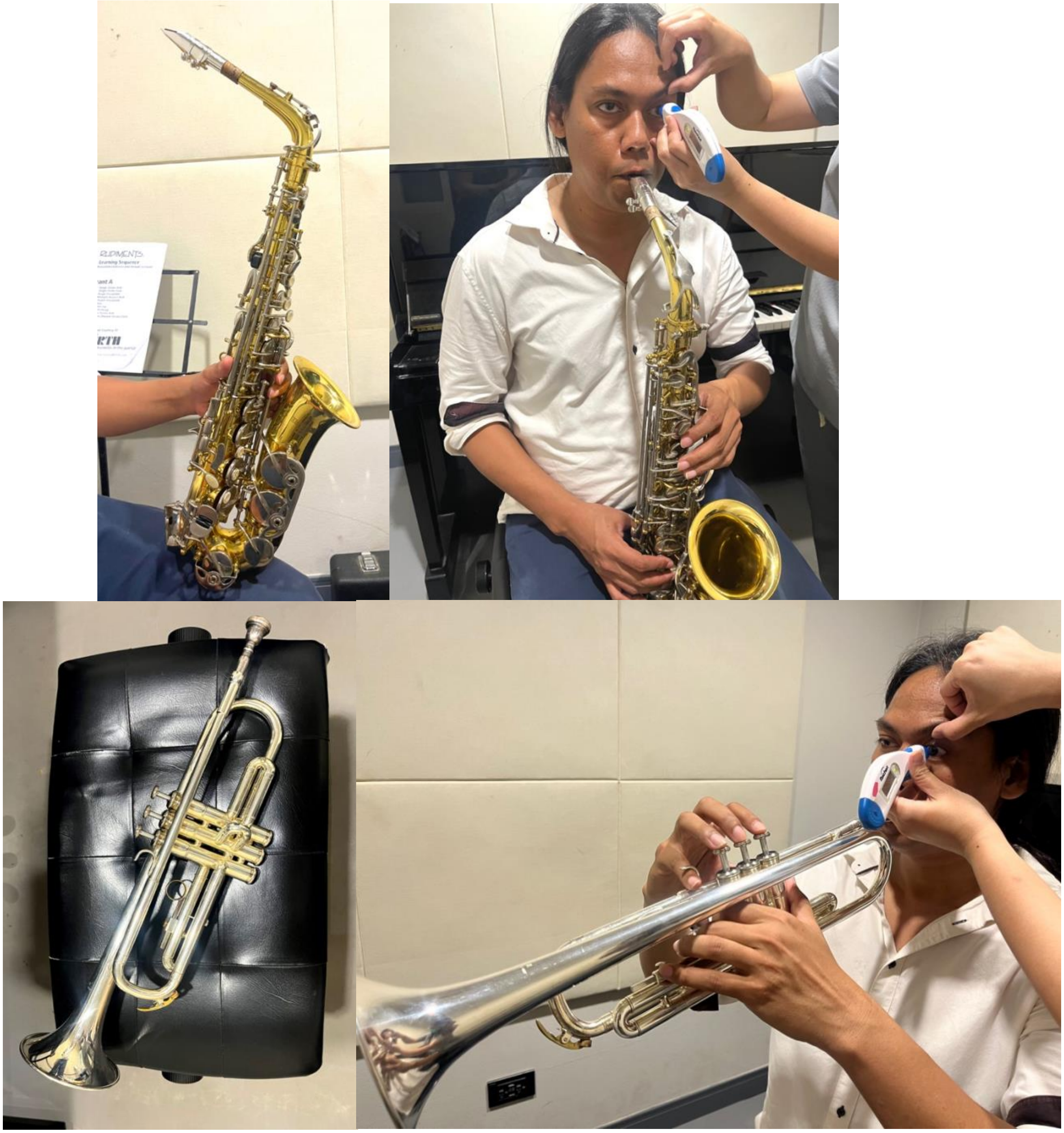

Group 2: Thai traditional (TT) wind instrument; Khlui

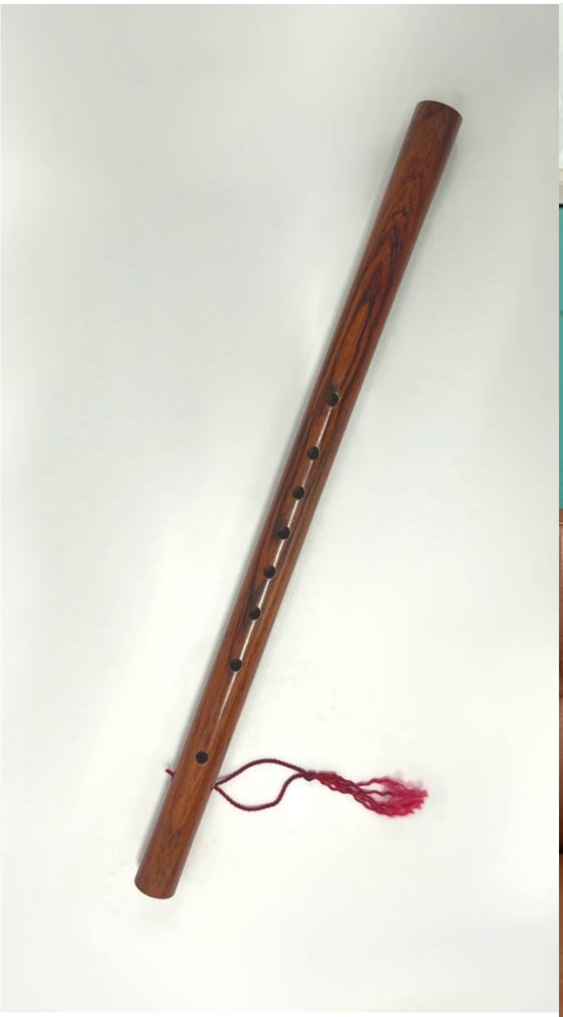

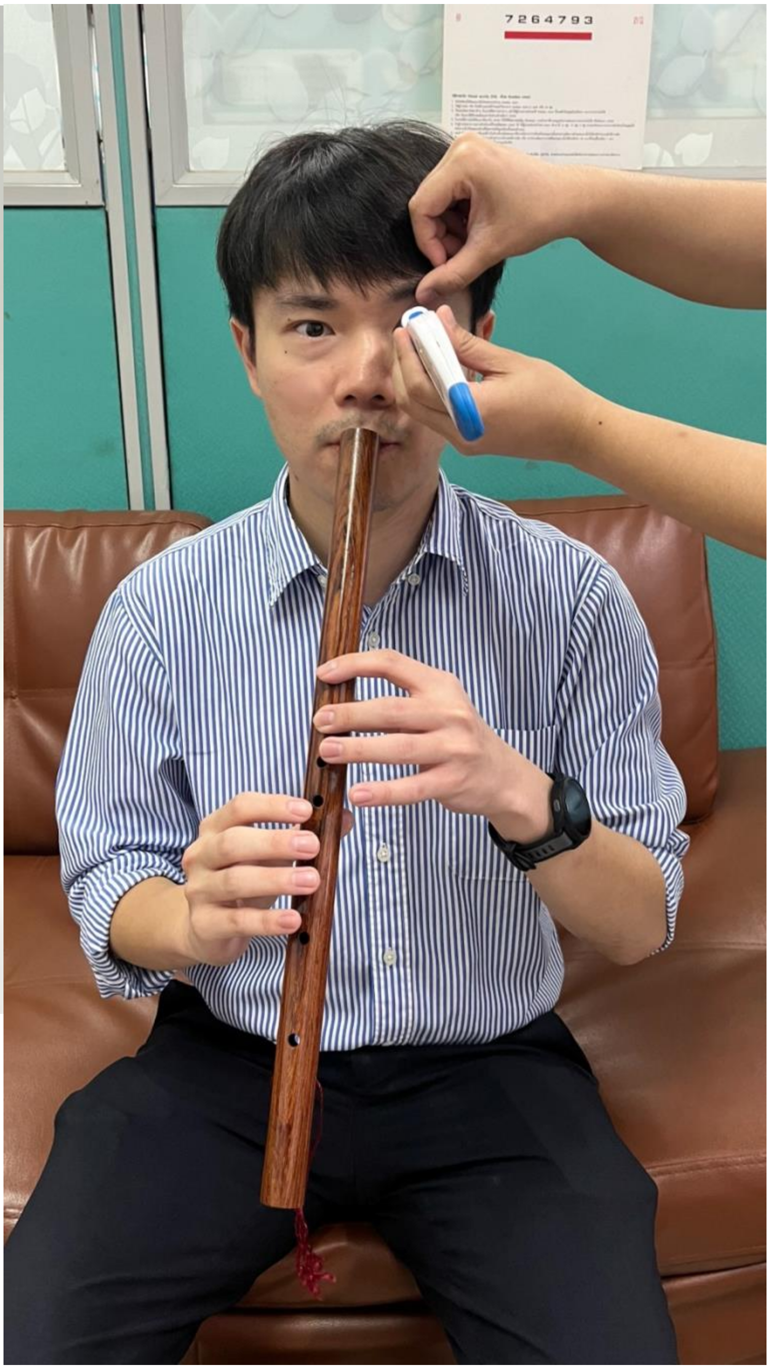

Group 3: Thai folk (TF) wind instrument; Khaen

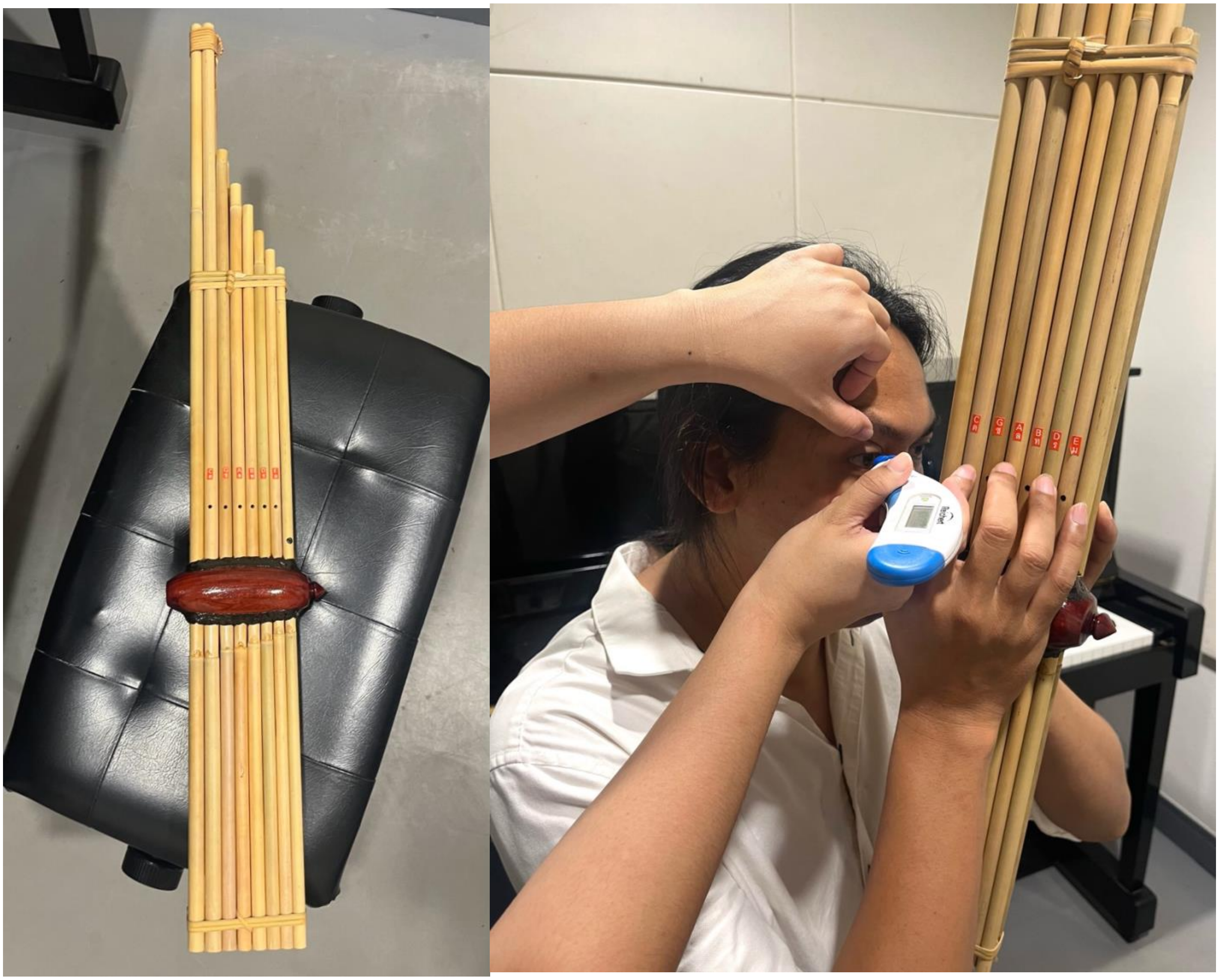

Note: These figures demonstrate the posture used during IOP measurement. The individuals shown are for illustrative purposes only; participants in each group are not the same person.
