# Supplementary material for: Changes in intraocular pressure before, during, and after playing Thai Traditional, Thai Folk, and Western wind instruments
Source: PLoS One. 2025 Oct 23;20(10):e0333533. doi: 10.1371/journal.pone.0333533 (PMC12548883; doi:10.1371/journal.pone.0333533)
Supplement: Attachment — Submitted filename: Response to Reviewers.pdf [file pone.0333533.s004.pdf]

REBUTTAL LETTER

Dear Reviewers,
Thank you for taking time to read and review our manuscript, which has greatly contributed to enhancing its scientific quality.

We have revised the manuscript and provided point-by-point responses as follow:

Reviewer #1

Response from reviewer:
1. The authors did not adjust for sex and age differences in their analyses; please re-do the analyses adjusting for these factors.

New/edit sentence in manuscript:
All the results in the manuscript text, Table 2 and Table 3 were updated.
Line 154
"The analysis was adjusted for age and sex covariates."

Explanation:
Thank you for your suggestion. We re-analyzed the data with adjustments for age and sex and found that the main outcome remained consistent with our previous report. The results in the manuscript text and all tables have been revised accordingly.

Response from reviewer:
2. The authors should acknowledge that the results being non-significant could be because the sample size was too small.

New/edit sentence in manuscript: -

Explanation:
Thank you for your concern regarding the sample size. As detailed in the Methods section, we conducted a sample size calculation based on mixed-effects model analysis using five repeated IOP measurements from both eyes. The estimated required sample size was five patients per group. Although the number of participants may appear small, all subjects contributed 120 data points in total. Based on this model, the calculated statistical power was 88%, with an alpha error

of 0.05, as reported in the manuscript. Furthermore, we have provided a document file demonstrating the sample size calculation using the R programming language, which includes details on the fixed and random effects applied to simulated data. In light of this, we believe the sample size is sufficient to support the validity of our analysis. We appreciate your thoughtful consideration.

Reviewer #2

Response from reviewer:
No information is provided about players' experience or skill levels, which may influence blowing technique and IOP changes.

New/edit sentence in manuscript
Line 168
"The mean (SD) duration of wind instrument experience was 3.9 (1.1) years, with no significant difference observed between groups (P=0.35)."

Please see Table 1 – Experience, years

Explanation:
Thank you for your valuable comments. We have included information on the musicians' experience by reporting the mean number of years of instrument practice, based on participants' self-report. Additionally, we have provided the years of experience for participants in each group in the revised Table 1.

Response from reviewer:
2. While using Tono-Pen during performance is understandable, the number of IOP measurements taken at each timepoint is unclear. Was there only one measurement per timepoint? The measurement error under dynamic conditions requires discussion, including intra-observer variability and comparison with standard methods e.g., GAT.

New/edit sentence in manuscript
Line 125
"All IOP measurements were performed by a single evaluator using one Tono-Pen AVIA® (Reichert, Inc., Depew, NY). According to the device's measurement technique, the average IOP with its 95% confidence interval were calculated and displayed after five taps (or ten measurements)."

Line 334
"Firstly, IOP measurement was performed using the Tono-Pen, as the use of Thai instruments obstructed the Slit-lamp examination and precluded the application of Goldmann applanation tonometry. It is important to note that the measurement errors may have occurred due to dynamic conditions during the performance period. Nevertheless, assessing ten consecutive IOP measurements with the Tono-Pen, as described, likely reduce intra-observer variability."

Explanation
We have added details on the number of IOP measurements and the use of the Tono-Pen in the Methods section, along with a brief discussion of its limitations.

Response from reviewer:
3. The player position (sitting/standing) during performance and IOP measurement is not specified, despite the known effect of posture on IOP. Address how breathing technique and posture differences between groups might influence IOP changes.

New/edit sentence in manuscript
Line 101
"The breathing technique for each instrument differed. For the saxophone and trumpet, participants used diaphragmatic (abdominal) breathing, inhaling quickly and deeply through the mouth. The breath fills the lungs, causing the waist to expand outward, and air was exhaled steadily and controlled by the diaphragm to produce a consistent tone. For the Khlui, participants also employed diaphragmatic breathing, inhaling through the nose and controlling the airflow with the diaphragm. The breath is then controlled and released through the mouth to produce sound. There is no use of cheek air storage or circular breathing. In contrast, the Khaen required the use of circular breathing. Participants inhaled through the nose while simultaneously pushing stored air from the cheeks into the instrument, allowing for continuous sound production. All musicians were seated during both the performance and IOP measurement. Documentation of each instrument and the participants' postures across all groups is provided in S2 Fig."

Explanation
We added a paragraph to address the breathing techniques associated with each instrument and the posture of the musicians. Supplementary document S2 has been attached to demonstrate the musicians' postures and the procedure used to obtain IOP measurements during performance.

Response from reviewer:
4. IOP measurements from right and left eyes should be separately analyzed. The percentage change at 37s should reference baseline IOP for right eyes, while change at 101s should reference baseline IOP for left eyes. Table 1 implicitly assumes equal mean IOP for both eyes in each group, which although possible, is unlikely.

New/edit sentence in manuscript
Line 154
"Fixed effects included group, timepoint (baseline, 37, 101, 150, 240 seconds), and baseline IOP. Additionally, second-matched baseline IOP and the interaction between timepoint and second-matched baseline IOP were included as covariates. A random intercept was used to account for potential inter-individual variability in baseline IOP between the right and left eyes."

Line 174
"Additionally, IOP measurements did not differ significantly between the right and left eyes within any group."

Please also see Table 1

Explanation
Thank you for your comment. We selected a mixed-effects model for this analysis. In accordance with the principles of mixed model analysis, intraocular pressures (IOPs) from both the right and left eyes of each individual were included, with the model accounting for within-subject correlation by specifying the eyes as random effects. We also employed a random intercept to account for potential differences in baseline IOP between individuals. We believe this method is appropriate and statistically justified. In response to reviewer's suggestion, we've added the IOP data for right and left eyes of participants in each group, as presented in Table 1 and revised the paragraph in the **statistical analysis subsection** to explain more about the fixed effect and random intercept that we used in the analysis.

Response from reviewer:
5. The terms "high-resistance" and "low-resistance" wind instruments need clearer definition for readers unfamiliar with wind-instrument acoustics.

New/edit sentence in manuscript
Line 47
"The wind instruments were categorized into high-resistance and low-resistance groups. High resistance wind instruments (HRWI), such as the trumpet and French horn, required greater breath pressure and lower airflow rates to produce sound. In contrast, low-resistance wind instruments, such as the saxophone and trombone, require less breath pressure and involve higher airflow rates."

Explanation
We added a paragraph in the **introduction section** to explain the difference between HRWI and LRWI.

Response from reviewer:
6. Images of the Khlui and Khaen would help readers understand these unfamiliar instruments. Better still, an image of a participant in each of the groups blowing their instrument while IOP is being recorded would be a valuable addition to the methods section.

New/edit sentence in manuscript
Line 112
"Documentation of each instrument and the participants' postures across all groups is provided in S2 Fig."

Line 134
"A demonstration of a participant from each group performing on their respective instrument during IOP measurement is presented in S2 Fig."

Explanation
Thank you for this valuable comment. We have attached a document in the method section that demonstrates the instruments and the participant's posture while IOP is being recorded.

Response from reviewer:
7. Combining saxophone and trumpet players into a single "Western" group is problematic given their different blowing techniques and resistance levels. Although this limitation was addressed in the discussion, data on specific Western instruments played would be valuable (e.g., how many people played the sax vs trumpet).

New/edit sentence in manuscript
Line 176
"In the Western group, three of the five musicians played the saxophone (two females, one male), and two played the trumpet (one female, one male). The saxophone and trumpet players had mean ages of 24.6 and 26 years, respectively, and mean playing experience of 4.6 and 4.5 years."

Explanation
Thank you for the suggestion. We have added details of the participants playing saxophone and trumpet in the result section.

Response from reviewer:
8. Expand discussion of clinical implications, particularly for musicians with pre-existing glaucoma or ocular hypertension.

New/edit sentence in manuscript
Line 316
"A previous study has shown that glaucoma patients exhibited higher mean IOP and greater IOP fluctuation after playing the high- and low-resistance wind instruments for 20 minutes, compared to individuals without glaucoma[9]. One case report described a glaucoma patient who experienced a markedly increase in IOP while playing the oboe. The IOP elevation was more pronounce in the eye without prior trabeculectomy, whereas the eye with advanced glaucoma and a functional trabeculectomy showed a smaller increase. Notably, IOP returned to baseline levels within five minutes of rest. Although our study was conducted in young, healthy musicians, we hypothesize that the transient IOP elevation may be more pronounced in musicians with pre-existing glaucoma or ocular hypertension, potentially due to impaired ocular autoregulation. This transient elevation could contribute to an increased risk of glaucoma progression in such individuals."

Explanation
Thank you for the insightful comment. We added paragraph in the discussion section.
