# Supplementary material for: Changes in intraocular pressure before, during, and after playing Thai Traditional, Thai Folk, and Western wind instruments
Source: PLoS One. 2025 Oct 23;20(10):e0333533. doi: 10.1371/journal.pone.0333533 (PMC12548883; doi:10.1371/journal.pone.0333533)
Supplement: Attachment — Submitted filename: Response_to_Reviewers_auresp_2.pdf [file pone.0333533.s005.pdf]

**Responses to reviewers**

Dear Reviewers,

Thank you for taking time to read and review our manuscript, which has greatly contributed to enhancing its scientific quality. We have revised the manuscript and provided point-by-point responses as follow:

**Reviewer #3:**

**Response from reviewer**

1. The only thing I would ask you to consider adding to your conclusion (which you have in the discussion) is the caution needed with glaucoma patients or glaucoma suspects.

**New/edit sentence in manuscript**

*Line 369*

"normal-tension glaucoma, high-tension glaucoma, or glaucoma suspects"

*Line 382*

"Nevertheless, caution should be warranted when interpreting these results in individuals with glaucoma or glaucoma suspects."

**Explanation**

Thank you for your suggestion. I have added the sentence at the end of the conclusion paragraph. Also, I've added the 'glaucoma suspects' in the limitation of the discussion part.

**Reviewer #4:**

**Response from reviewer**

1. The use of a linear mixed-effects model and simulation-based sample size estimation is methodologically appropriate, the inclusion of only 15 participants may raise concerns regarding the stability and generalizability of the model estimates. In models with multiple timepoints, fixed and random effects, and potentially correlated within-subject measures, a larger number of subjects is typically advisable to ensure robust variance estimation and avoid overfitting.

**New/edit sentence in manuscript**
*Line 362*
"Additionally, the sample size of five participants per group (total n =15) is relatively small. Although this sample size achieved a statistical power of 0.88 to detect differences in IOP among the three groups, caution is warranted because this limited number might not fully capture inter-subject variability. Furthermore, there is a possibility that the effect size from the simulated data that used for power calculation was overestimated, potentially leading to an underestimation of the required sample size"
*Line 372*
"larger sample sizes"

**Explanation**
Thank you for your valuable comment. We have incorporated a statement regarding the sample size caution in the limitations section of the manuscript and also added the suggestion for the future study.

**Response from reviewer**
2. Discussion section, paragraph 1, In page no. 15 line no. 263-265: The claim of being the first study in this area should be made with caution. A more cautious phrasing, such as "this appears to be a relatively unexplored area…," would be more appropriate and academically balanced.

**New/edit sentence in manuscript**
*Line 271*
"To our knowledge, this appears to be a relatively unexplored area, particularly regarding IOP changes associated with playing Thai traditional and Thai folk wind instruments."

**Explanation**
Thank you so much. We have revised the sentence as you suggested.

**Reviewer #5:**

**Response from reviewer**
1. It would be advisable to ensure consistency between the Results and Conclusions sections of the abstract. If the conclusion states that "Elevated IOP was demonstrated during the playing of three types of wind instruments," then this

finding should also be explicitly presented in the Results section. Furthermore, since the primary outcome was the difference in IOP between the groups, it would enhance clarity to indicate in the abstract whether there were statistically significant differences in baseline IOP among the three groups.

**New/edit sentence in manuscript**

*Line 30 in abstract*

Thirty eyes from 15 participants were included, with mean (SD) baseline IOP of 13.3 (2.7), 12.3 (1.5), and 14.5 (2.4) mmHg in WT, TT, and TF groups ($P = 0.53$).

*Line 34 in abstract*

During the performance (from baseline to 101 seconds), IOP increased significantly in WT (3.1, 0.6 to 5.7, 0.007) and TT groups (2.8, 0.2 to 5.3, 0.02), while TF group did not show a significant increase. However, no significant differences were found when comparing among the three groups. After the performance (150 seconds), mean IOP differences between groups were small and did not show statistically significant.

*Line 40 in abstract*

"Conclusions: The three types of wind instruments demonstrated no significant differences in IOP elevation during or after performance. While a transient increase in IOP was observed during playing in all groups, IOP returned to baseline within 30 seconds after the performance"

*Line 375*

"Conclusion

No significant differences in IOP were observed before, during or after playing Thai traditional, Thai folk, or Western wind instruments. Although a mild and transient elevation in IOP was noted during performance, IOP levels returned to baseline within 30 seconds after performance was stopped in all groups. These findings suggest that playing Thai and Western wind instruments for a short period of time should not be a concern for increasing the risk of glaucoma in young, healthy individuals. Nevertheless, caution should be warranted when interpreting these results in individuals with glaucoma or glaucoma suspects."

**Explanation**

Thank you for your valuable comments. We rearranged the conclusions in the abstract and manuscript to make them more consistence with the primary outcome.

**Response from reviewer**
2. Although the sample size was calculated to include five participants per group, based on a power analysis, a total of 15 participants is a relatively small sample size. This raises concerns that inter-subject variability may not be adequately captured. It is also possible that the effect size used in the simulation was overestimated, leading to an underestimation of the required sample size. These limitations should be acknowledged in the manuscript to allow readers to appropriately interpret the findings.

**New/edit sentence in manuscript**
*Line 362*
"Additionally, the sample size of five participants per group (total n =15) is relatively small. Although this sample size achieved a statistical power of 0.88 to detect differences in IOP among the three groups, caution is warranted because this limited number might not fully capture inter-subject variability. Furthermore, there is a possibility that the effect size from the simulated data that used for power calculation was overestimated, potentially leading to an underestimation of the required sample size"

**Explanation**
Thank you for your valuable comment. We have incorporated a statement regarding the sample size caution in the limitations section of the manuscript.

**Reviewer #6:**

**Response from reviewer**
1. Due to the consideration of gender as a factor affecting IOP changes during performance, the author should be recommended to explain whether baseline data will impact the study results in the Thai folk group, where all performers are male, compared to the other two groups.frequency

**New/edit sentence in manuscript**
*Line 308*
"…and sex."
*Line 313*
"Notably, the TF group consisted entirely of male participants, whereas the other groups included both genders. While no statistically significant difference in sex distribution was observed among groups (P = 0.08), potential sex-related effects on

IOP warrant cautious interpretation [13]. Therefore, sex was included as a covariate in the statistical analysis to adjust for its potential confounding effect."

**Explanation**
Thank you for your valuable suggestion about this issue. We acknowledge that gender may act as a confounding factor, hence, we included gender as a covariate in the analysis to adjust for its potential effect. This condiseration has been incorporated into the discussion part.

**Response from reviewer**
2. The authors should acknowledge that the results being non-significant could be because participants played the low tone frequency and low playing intensity musical notations, and further supplement studies data with higher strength notes

**New/edit sentence in manuscript**
*Line 302*
"These low-intensity settings might also explain the nonsignificant differences in IOP elevation between groups. Further studies employing higher tone frequencies and increased playing intensity might help determine whether these factors affect IOP changes across different instrument groups."

**Explanation**
Thank you. We have discussed more about the cause of nonsignificant IOP change between groups (which is the primary outcome of the study) and proposed the idea for further studies.

**Response from reviewer**
3. The authors did not adjust for the effect of CCT on IOP in their analyses; please redo the analyses after Correction value.

**New/edit sentence in manuscript**
*Line 135*
"CCT-adjusted IOP values were calculated using the online IOP calculator [7]. These corrected IOP values were subsequently used for the analysis."
*Line 177*
"CCT was higher in the Thai traditional group compared to the others, therefore, CCT-adjusted IOP value was calculated. Corrected IOP values of the right and left eyes did not differ significantly between groups, nor between eyes within any group."

All outcome values in the manuscript
Table 1: adjusted IOP
Table 2,3: all data
Figure 1A, 1B

**Explanation**
Thank you for your valuable comment. We performed the analysis again using the corrective value of IOP and found that the new result was consistent with our previous findings. We reported the corrective value and the new outcome in Table 1-3 and Fig1A,1B (the spaghetti plot). The corrective value was calculated by IOP-CCT correction formula referenced by https://www.iop-calculator.com/.

***************

We also revised some sentences to ensure clarity in the discussion section.

Line 292
It can be concluded that Thai wind instruments cause a temporary IOP rising comparable to that observed with Western wind instruments, despite differences in playing and breathing techniques.

Line 325
We assume that this rapid return of IOP after the performance may be less efficient in elderly musicians or those with glaucoma. This is likely due to ischemic optic nerve damage associated with aging and glaucoma, which can impair ocular autoregulation [15]. Consequently, these individuals may experience delayed or inadequate IOP normalization following elevated IOP episodes.

***************
