# Supplementary material for: Changes in intraocular pressure before, during, and after playing Thai Traditional, Thai Folk, and Western wind instruments
Source: PLoS One. 2025 Oct 23;20(10):e0333533. doi: 10.1371/journal.pone.0333533 (PMC12548883; doi:10.1371/journal.pone.0333533)
Supplement: Attachment — Submitted filename: Response_to_Reviewers_auresp_3.pdf [file pone.0333533.s006.pdf]

Responses to reviewers

Dear Reviewers,
Thank you for taking time to read and review our manuscript, which has greatly contributed to enhancing its scientific quality. We have revised the manuscript and provided point-by-point responses as follow:

1.
Response from reviewer
Abstract: line 39 is incomplete; change to "were not significantly different".

New/edit sentence in manuscript
Line 39
"After the performance (150 seconds), mean IOP differences between groups were small and were not significantly different."

Explanation
Thank you for your suggestion. I have revised the sentence.

2.
Response from reviewer
Abstract: line 43 needs a period.

New/edit sentence in manuscript
Line 43
"While a transient increase in IOP was observed during playing in all groups, IOP returned to baseline within 30 seconds after the performance."

Explanation
Thank you. I added a period.

3.
Response from reviewer
Throughout the text, please do not use the term "elderly" as that is considered a negative descriptor; instead substitute with "older"

New/edit sentence in manuscript
Line 325
"...in the older. . We assume that this rapid return of IOP after the performance may be less efficient in older musicians or those with glaucoma."

Explanation
Thank you so much. We have revised the sentence as you suggested.

4.
I have reviewed the reference articles as the journal suggested. All are complete and correct. However, I changed the German title of one reference article to its English version for consistency.

New/edit sentence in manuscript
Line 389
1. Kappmeyer K, Lanzl IM. Intra-ocular pressure during and after playing high and low resistance wind instruments. Ophthalmologe. 2010;107:41–6.

5.
According to the question below, I have published the repository data for this research on OSF. Please go to the 'Files' tab first and the document will be displayed (as demonstrated below).

> *Q: Have the authors made all data underlying the findings in their manuscript fully available?*
>
> *The PLOS Data policy requires authors to make all data underlying the findings described in their manuscript fully available without restriction, with rare exception (please refer to the Data Availability Statement in the manuscript PDF file). The data should be provided as part of the manuscript or its supporting information, or deposited to a public repository. For example, in addition to summary statistics, the data points behind means, medians and variance measures should be available. If there are restrictions on publicly sharing data—e.g. participant privacy or use of data from a third party—those must be specified.*
>
> *Reviewer #1: No*
>
> *Reviewer #5: Yes*

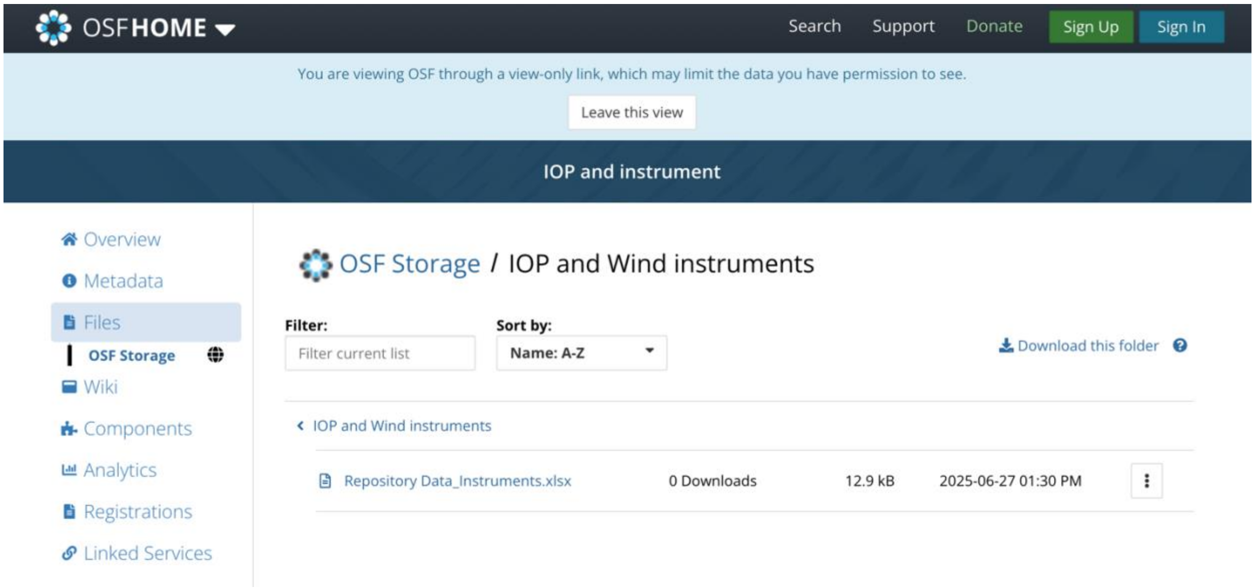
OSFHOME
Search
Support
Donate
Sign Up
Sign In
You are viewing OSF through a view-only link, which may limit the data you have permission to see.
Leave this view
IOP and instrument
Overview
Metadata
Files
OSF Storage
Wiki
Components
Analytics
Registrations
Linked Services
OSF Storage / IOP and Wind instruments
Filter:
Filter current list
Sort by:
Name: A-Z
Download this folder
IOP and Wind instruments
Repository Data_Instruments.xlsx
0 Downloads
12.9 kB
2025-06-27 01:30 PM

****
